# Transcriptomic Analysis of *Pediococcus pentosaceus* Reveals Carbohydrate Metabolic Dynamics Under Lactic Acid Stress

**DOI:** 10.3389/fmicb.2021.736411

**Published:** 2021-09-16

**Authors:** Dong Han, Qiaojuan Yan, Jun Liu, Zhengqiang Jiang, Shaoqing Yang

**Affiliations:** ^1^Key Laboratory of Food Bioengineering (China National Light Industry), College of Food Science and Nutritional Engineering, China Agricultural University, Beijing, China; ^2^School of Food and Health, Beijing Technology and Business University, Beijing, China; ^3^College of Engineering, China Agricultural University, Beijing, China

**Keywords:** *Pediococcus pentosaceus*, lactic acid stress, carbohydrate metabolism, functional oligosaccharide, RNA-seq

## Abstract

Stress physiology of lactic acid bacteria (LAB) is crucial to their ecological fitness and applicational implications. As a self-imposed stress, lactic acid is the major final metabolic product of LAB and its accumulation can be detrimental to bacterial cells. However, the relationship between LAB carbohydrate metabolism, the primary energy supplying bioactivities, and lactic acid stress responses is not fully understood. *Pediococcus pentosaceus* has been recognized as an important cell factory and demonstrated probiotic activities. This study investigated behavior of *P. pentosaceus* under lactic and acetic acid stresses, particularly with supplementations of metabolizable carbohydrates. Lactic and acetic acid retain similar growth stagnation effect, and both resulted in cell death in *P. pentosaceus*. All metabolizable carbohydrates improved bacterial survival compared to lactic acid control, while xylooligosaccharides (XOS) exerted the highest viability protective efficacy, 0.82 log CFU/mL higher population survived than other carbohydrates after 30 h of incubation. RNA-seq pipeline showcased the intensive global transcriptional responses of *P. pentosaceus* to lactic acid, which caused significant regulations (more than 2 Log_2_ fold) of 16.5% of total mRNA coding genes. Glucose mainly led to gene suppressions (83 genes) while XOS led to gene up-regulations (19 genes) under lactic acid stress. RT-qPCR study found that RNA polymerase-centered transcriptional regulation is the primary regulatory approach in evaluated culture conditions. The synergy between lactic acid stress and carbohydrate metabolism should be attentively contemplated in future studies and applications.

## Introduction

Traditionally, the applications of lactic acid bacteria (LAB) have been primarily associated with the production of fermented foods, such as vegetables, meats, dairies, and beverages (Zannini et al., 2016). Moreover, modern biotechnologies and bioengineering have transformed many LAB species, e.g., *Lactococcus lactis* (Song et al., 2017) and *Lactiplantibacillus plantarum* (Mathiesen et al., 2008), into cell factories to produce bioactive molecules (van Tilburg et al., 2019). Stress physiology of LAB has been extensively studied to improve related industrial practices as well as to expand the knowledge in bacteriology. Understandably, as a self-imposed stress, acidic stress is often gradually introduced to the bacterial culture in company with the proliferation of LAB and the accumulation of lactic acid (LA). These altered culture environments are likely harmful to LAB and could eventually stagnate culture growth and related bacterial activities (Liu et al., 2015; Papadimitriou et al., 2016). Therefore, the utilization of LAB in both fermented food production and engineered industrial fermentation requires robust acid tolerance in featured bacteria strains (Wu et al., 2014). The connection between acidic resistance of LAB and the presence of metabolizable carbohydrates have been previously established. Previous research (Corcoran et al., 2005) demonstrated that the presence of metabolizable carbohydrates could enhance the survival of Lactobacilli, although different LAB strains displayed different level of survival enhancement with the supplementation of glucose. Another study (Gullón et al., 2014) showcased the improvement in acidic stress resistance of targeted LAB with different xylooligosaccharides (XOS) which possess potential for symbiotic product development. Also, since the broad adoption of bioinformatic methods, many studies verified that LAB could exert probiotic activities to improve overall host gut health and combat chronic diseases (Toumi et al., 2014; Pandey et al., 2015; Saez-Lara et al., 2015). Notably, colonization of LAB relies on their passage through upper gastrointestinal (GI) track to reach lower GI track in viable state, thus it is necessary for LAB to survive extreme acidic gastric environment to manifest their probiotic functionalities (Bezkorovainy, 2001; Klu and Chen, 2015). In summary, acid tolerance of LAB is of great importance to their applications.

*Pediococcus pentosaceus* is a gram-positive LAB belongs to the family of *Lactobacillaceae*. It has been recognized as an important species in fermented foods, such as fermented beverage, meats, vegetables, dairy products, and wine (Todorov and Dicks, 2005; Shin et al., 2008; Osmanagaoglu et al., 2010; Vidhyasagar and Jeevaratnam, 2013). *P. pentosaceus* was also proven to possess host health improvement effects, like producing beneficial bacteriocin (Porto et al., 2017) and cholesterol decreasing effect (Ilavenil et al., 2016). Whole genome sequencing of *P. pentosaceus* has been previously conducted and the study revealed that *P. pentosaceus* is closely related phylogenetically to members of the *Lactobacillaceae* family, such as *Lactiplantibacillus plantarum* and *Levilactobacillus brevis* (Makarova et al., 2006). Universal transcriptome sequencing analysis revealed that functional oligosaccharide culture environments, such as fructooligosaccharides (FOS), konjac mannooligosaccharides (KMOS), and XOS, could systematically regulate the global gene expression of *P. pentosaceus* and extensively change the transcriptomic profile (Han et al., 2021). This study demonstrated these functional oligosaccharides could up-regulated acid tolerance-related gene clusters in *P. pentosaceus*, such as arginine deiminase system.

Here, these functional oligosaccharide supplementations were comprehensively evaluated on viable population growth and survival of *P. pentosaceus* under LA or acetic acid (AA) environments. Then global transcriptome sequencing and follow-up gene expression analysis was employed to explore underlying gene expression mechanisms.

## Materials and Methods

### Bacterial Strains and Carbohydrates

*P. pentosaceus* CGMCC 1.7665, *P. pentosaceus* CGMCC 1.2441 (equal to ATCC 33,314), and *P. pentosaceus* CGMCC 1.10,999 were incubated in de Man, Rogosa and Sharpe (MRS) broth (BD Difco, Sparks, MD, United States) to an optical density value at 600 nm (OD_600_) of 1.0 and stored at −80°C with the supplementation of 20% glycerol. To prepare precultures, frozen strains were incubated in MRS broth at 37 °C for 18 h and then refrigerated at 4°C prior to further analysis. Investigated carbohydrates were acquired from respective suppliers: glucose (Amresco, Solon, OH, United States), xylose (Alfa Aesar, Ward Hill, MA, United States), FOS (Orafti^®^ P95, Orafti Active Food Ingredients, Tienen, Belgium), XOS (95P, Longlive Biotechnology, Dezhou, China), and KMOS (Xi’an Yuansen Biological Technology, Xi’an, China).

### Culture Conditions and Bacterial Cell Density Monitoring

Modified MRS (mMRS, formulation listed in Supplementary Table 3) media were prepared by suppling 2% (w/v) different saccharides into MRS cultures prior to autoclave sterilization at 121°C and 15 psi for 20 min. To achieve organic acid stress environments, pH adjustment was carried out by gradually adding 10% (v/v) sterilized lactic acid or acetic acid into post-sterilization media with continuous vortex homogenization until certain pH level (6, 5, or 4.2) was reached. Strains for formal experiments were prepared by transferring 100 μL refrigerated culture into 10 mL uninoculated MRS media and incubated overnight at 37°C. Overnight bacterial culture was washed twice and adjusted to OD_600_ of 1.0 using sterilized 0.85% NaCl saline solution to prepare the inoculum. To be noticed that all the surveyed cultures in this study were grown without controlled pH, which means pH values were correspond to different media and may decrease at different paces during growth. For growth monitoring, the inoculum was transferred into pH adjusted mMRS media in a 1:40 ratio between the total volume of inoculum and final bacterial culture. After inoculation, 200 μL of bacterial culture was transferred into individual wells of 96-well plates and covered by 50 μL sterilized mineral oil (Sigma Chemical Co., St. Louis, MO, United States) to create anaerobic culture conditions. A 96-well plates OD UV reader (multiskan FC, Thermo Electron Corporation, Waltham, MA, United States) was employed to record cell optical density values at 595 nm wavelength (OD_595_) during the 30 h of incubation. Changes of OD_595_ (calculated by subtracting OD_595_ at 0 h from OD_595_ recorded at each sampling time) were plotted to monitor cell densities.

### Remaining Population Measured by Plate Counting

In this section, 1.5 mL of inoculated mMRS media with pH adjustments were incubated in 2 mL Eppendorf tube and incubated in an anaerobic incubator (LAI-3T, Shanghai Longyue Instrument Equipment Co., Shanghai, China) at 37°C. Remining culturable population of each sample was enumerated by plating decimal serial dilutions of 100 μL culture.

### mRNA Extraction and RNA-Seq

Inoculum prepared from previous section (OD_600_ = 1.0) was centrifuged at 10,000 rpm and 4°C for 2 min. After supernatant was completely removed, the pellet was mixed with mMRS media of which was adjusted with lactic acid to pH 4.2. After 45 min of anaerobic incubation, transcriptional activities of the bacterial culture samples were terminated by adding 1:1 pre-chilled isopropanol. Experiment kits, including RiboPure^TM^ RNA Purification Kit for bacteria (Ambion, Austin, TX, United States), Ribo-Zero^TM^ rRNA Removal Kit (bacteria) (Ambion, Austin, TX, United States), and DNase I digestion kit (Takara Ltd, Tokyo, Japan), were used for RNA isolation, rRNA removal, and gDNA removal, respectively, of prepared culture samples. Finally, Illumina HiSeq X Ten platform (Illumina Inc., San Diego, CA, United States) was employed for RNA-seq.

### Transcriptome Analytical Pipeline

Overall quality control was carried out on Galaxy platform^1^ (Blankenberg et al., 2010) using FastQC (Andrews, 2010) while Trimmomatic (Bolger et al., 2014) was used for sequence filtering. Genome sequences of *P. pentosaceus* SL4 (NCBI assembly, ASM49626v1) was employed as reference for sequence alignment using Bowtie2 (Langmead and Salzberg, 2012). Overall analysis and virtualization were carried out using packages of HTSeq (Anders et al., 2015), DESeq2 (Love et al., 2014), and Shinycircos (Krzywinski et al., 2009; Yu et al., 2018). Then, functions and metabolic pathways involved for genes for interest were reconstructed according to Kyoto Encyclopedia of Genes and Genomes and Enzyme Commission numbers assigned in database of the Integrated Microbial Genomes and Microbiomes (IMG/M)^2^ and virtualized using iPath^3^ (Darzi et al., 2018).

### Gene Expression Analysis Using RT-qPCR

Total RNA was isolated for samples that were prepared following the same procedure employed by RNA-seq experiments, except more time points (15 min, 45 min, and 90 min) were subjected for RT-qPCR analysis. Here, cDNA was synthesized using PrimeScript^TM^ RT Master Mix (Takara Ltd., Tokyo, Japan). Primers for 15 emphasized genes (Supplementary Table 4) was designed using NCBI Primer-BLAST tools.^4^ And reverse-transcription quantitative real-time PCR (RT-qPCR) was carried out on CFX Connect^TM^ real-time PCR platform (Bio-Rad, CA, United States). Gene expression level was calculated following the ΔΔ*C*_T_ method: ΔΔ*C*_T_ = (*CT* target gene − *CT* 16S rRNA) sampling point − (*CT* target gene − *CT* 16S rRNA) control at 0 min.

### Statistical Analysis

At least three biological repeats were carried out for every experiments. Single-factor ANOVA were implemented using a SPSS software package (SPSS Inc., Chicago, IL, United States). Kolmogorov-Smirnov test were carried out to determined data normality. Then comparisons between two groups were performed using either Student’s *t*-test or Mann-Whitney *U*-test accordingly.

## Results

### Bacterial Cell Density and Remaining Population

**FIGURE 1:**
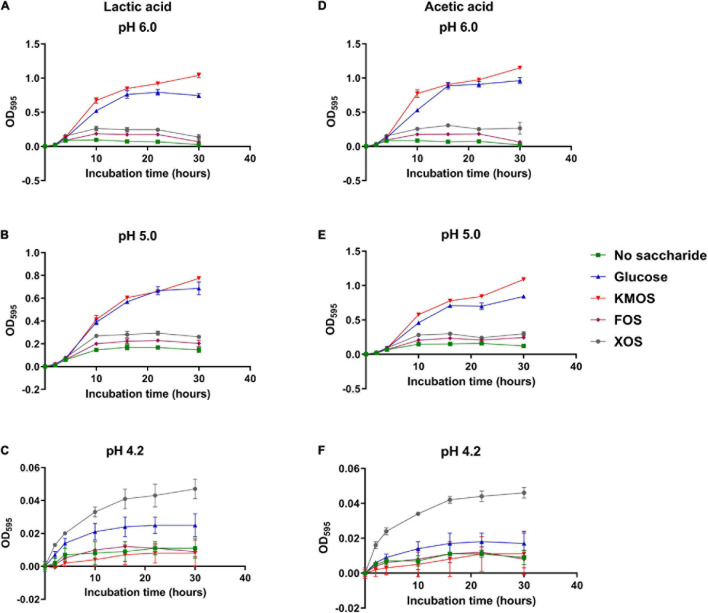
Growth kinetic of *P. pentosaceus* CGMCC 1.7665 in mMRS. Sterilized culture was adjusted using lactic acid to pH 6 **(A)**, 5.2 **(B)**, or 4.2 **(C)** or using acetic acid to pH 6 **(D)**, 5.2 **(E)**, or 4.2 **(F)**. Error bar represented standard deviation (*n* = 4).

Firstly, different carbohydrates were supplemented in modified mMRS broth and the culture pHs were adjusted using sterilized LA or AA. After inoculation of *P. pentosaceus* CGMCC 1.7665, optical density was monitored at OD_595_ during the 30 h incubation (Figure 1). Culture at initial pH 6.0 and 5.0 (Figures 1A,B,D,E) were not apparently different from each other as glucose and KMOS cultures led to higher population increase compared to other groups. However, culture of pH 4.2 significantly suppressed the OD_595_ growth in both LA (Figure 1C) and AA cultures (Figure 1F). In these culture conditions, all the groups generated minimal OD_595_ increase while only XOS cultures could result in a OD_595_ increases that were higher than 0.04, more than double to any other groups.

**FIGURE 2:**
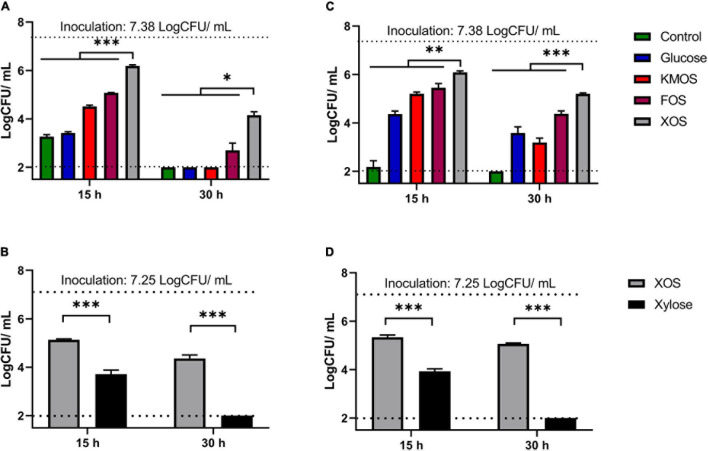
Remaining population for *P. pentosaceus* CGMCC 1.7665 measured by plate count method. Sterilized culture was adjusted using lactic acid **(A,B)** or using acetic acid **(C,D)**. (^∗^*P* < 0.05; ^∗∗^*P* < 0.01; ^∗∗∗^*P* < 0.001). Error bar represented standard deviation (*n* = 3). Detect limitation: 2 log CFU/mL.

Then, plate count method was carried out to evaluate the population in LA cultures (Figures 2A,B) as well as on AA cultures (Figures 2C,D). Result demonstrated that XOS supplied organic stress environment at pH 4.2 resulted in at least 0.82 log CFU/mL higher (*P* < 0.05) remaining culturable population compared to any other groups. This finding demonstrated XOS supplementations slowed down the culturable population decrease in both LA and AA stresses. While follow-up culture experiment between XOS and xylose cultures (Figures 2B–D) indicated that xylose could not match the population protective effect of XOS in either organic acid. Similar population reduction patterns among all the carbohydrate groups were also observed in two other *P. pentosaceus* strains (CGMCC 1.2441 and 1.10999) (Supplementary Figures 1, 2).

### Global Transcriptome Sequencing

**FIGURE 3:**
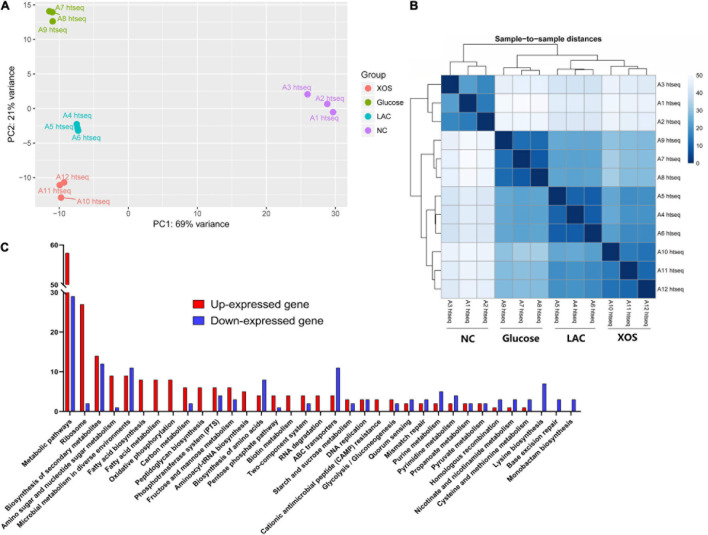
Comprehensive analysis of RNA-seq. Principal component analysis (PCA) plotting for 4 groups based on normalized overall transcriptomic reads **(A)**. Heatmap of 12 individual samples based on overall sample to sample distances in gene expression **(B)**. Significantly up- or down-expressed gene numbers in each pathway for LAC compared to NC according to Kyoto Encyclopedia of Genes and Genomes (KEGG) **(C)**, gene expression changes higher than 1.5 Log_2_ fold and −5 Log_10_ (*P*-value) was selected. NC, neutral control without carbohydrate supplement; LAC, lactic acid control of pH 4.2; Glucose, LAC supplied with glucose; XOS, LAC supplied with XOS.

Using RNA-seq, comprehensive transcriptomic responses of *P. pentosaceus* was carried out. In the Principal Component Analysis (PCA) (Figure 3A), the first two principle components (PC1 and PC2) that account for 90% of the cumulative reliabilities were plotted for all four groups. It can be inferred from the plot that highly repeatable transcriptional profiling results were documented using the RNA-seq pipeline. While clear separation between LA control (LAC), LA + glucose, and LA + XOS were observed in PCA plot, the most outstanding group-to-group difference were detected between neutral control (NC) and LA groups. This observation could also be made in sample-to-sample heatmap (Figure 3B) that indicates LA stress should be considered as a predominant transcriptional regulation factor in *P. pentosaceus*.

Then, highly regulated genes between NC and LAC groups were summarized according to Kyoto Encyclopedia of Genes and Genomes (KEGG). As shown in Figure 3C, metabolic pathways were heavily regulated in LAC compared to NC, in which 58 genes were up-expressed and 29 genes were down-expressed. Notably, also from a global perspective (Supplementary Figure 3), ribosome, biosynthesis of secondary metabolites, amino sugar and nucleotide sugar metabolism, and microbial metabolism in diverse environments were among the up-regulated functional pathways, whereas biosynthesis of secondary metabolites, microbial metabolism in diverse environments, biosynthesis of amino acids, ABC transporters, and lysine biosynthesis were detected as the down-regulated pathways.

### Gene Expressions

**FIGURE 4:**
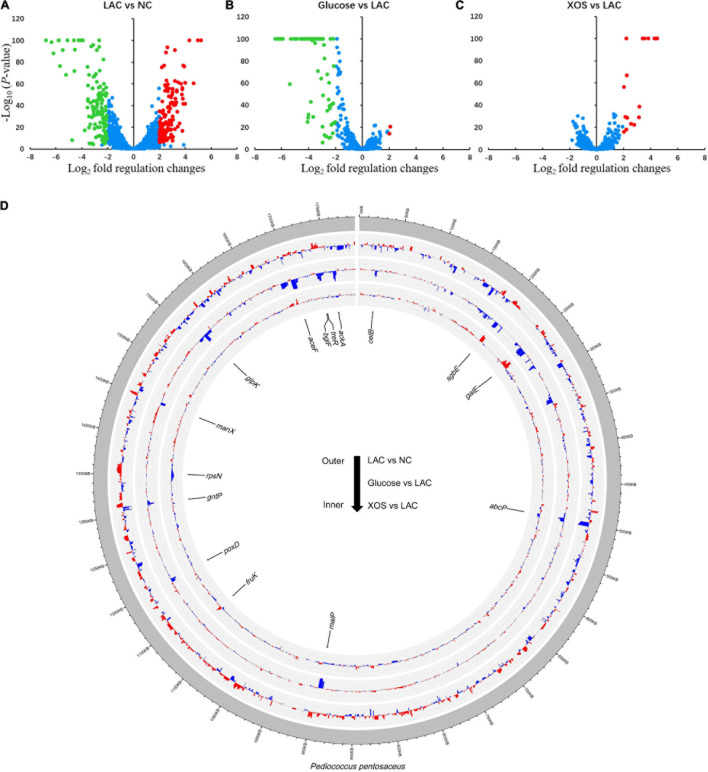
Global gene expression analysis. Log ratio and mean average (MA lotting) between each group: LAC versus NC **(A)**, Glucose versus LAC **(B)**, XOS versus LAC **(C)**. Genes expression changes that are higher than 2 log2-fold change and 5 log_10_ (*p*-value) are highlighted in red or green. Circos plot of global transcriptional variations for each comparison **(D)**. Gene expression value in former group compared to latter group is displayed using a positive value. Higher gene expression levels are displayed with red bars and lower expression levels are displayed with blue bars. Radial axis for each group represents expression differences ranged between−6 and 10 log2 (expression level).

In comparisons between individual groups (Figure 4), using 2 Log_2_ fold and −5 Log_10_ (*P*-value) as discrimination threshold, 129 genes were considered as up-regulated while 130 genes were significantly down-regulated in LAC, compared to NC (Figure 4A). In contrast, glucose displayed inferior regulation effects under LA stress whereas 2 and 83 genes were up- or down-regulated, respectively, compared to LC (Figure 4B). While 19 and 0 genes were up- or down-regulated in XOS group, respectively, compared to LAC (Figure 4C). These transcriptional regulations were delineated in Circos plot in which 15 functional genes were selected and highlighted for further analysis (Figure 4D).

### Metabolic Pathway Regulations

**FIGURE 5:**
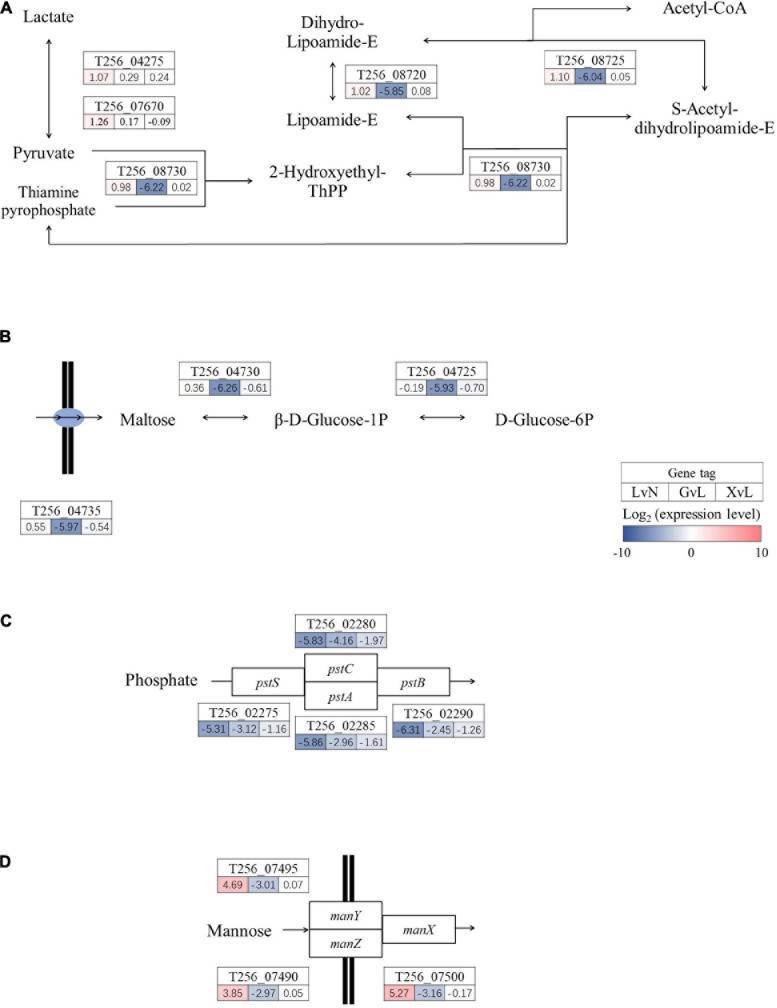
Significantly regulated functional metabolic pathways in *P. pentosaceus*. Pyruvate metabolism **(A)**, maltose metabolism **(B)**, phosphate ABC transporter pstSCAB **(C)**, and mannose transporter manXYZ **(D)** LvN, lactic acid group versus neutral group; GvL, Glucose group versus lactic acid group; XvL, XOS groups versus lactic acid group.

**FIGURE 6:**
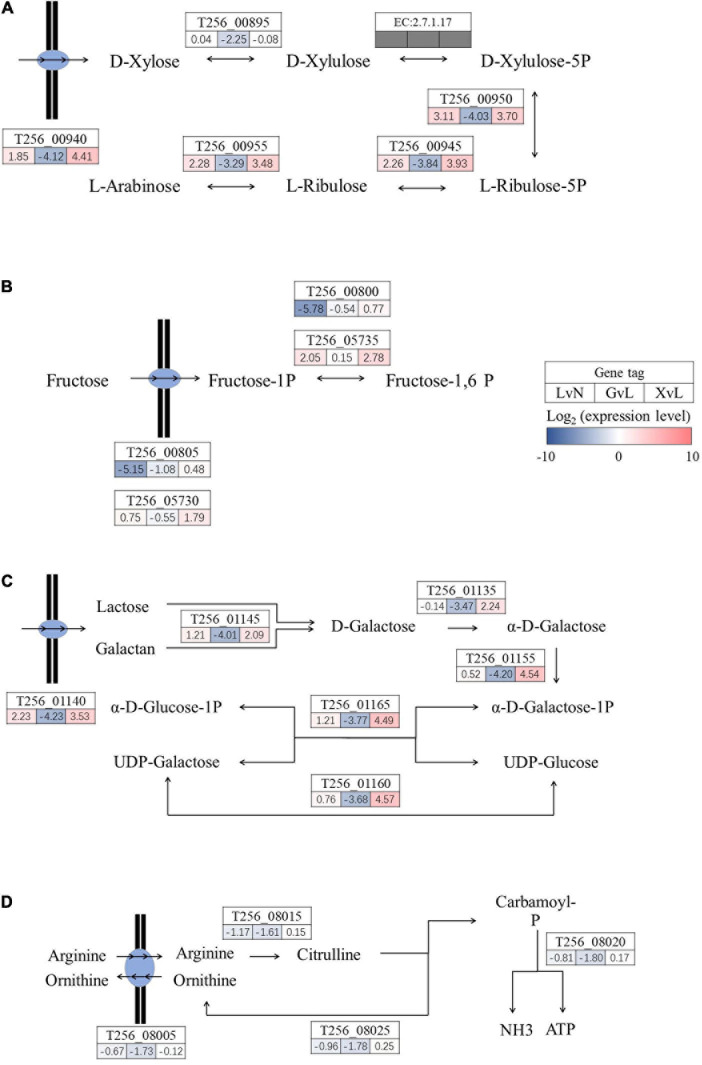
Significantly regulated functional metabolic pathways in *P. pentosaceus*. Xylose isomerization **(A)**, glycolysis components **(B)**, galactose metabolism **(C)**, and arginine deiminase system **(D)**. LvN, lactic acid group versus neutral group; GvL, Glucose group versus lactic acid group; XvL, XOS groups versus lactic acid group.

Moving on, as illustrated in Figures 5, 6, significantly regulated comprehensive microbial pathways were virtualized according to NCBI gene coding sequence locus tags. Among these pathways, pyruvate metabolism (Figure 5A) and maltose metabolism (Figure 5B) were dramatically down-regulated by glucose but not by XOS. And phosphate ABC transporter *pstSCAB* (Figure 5C) were down-regulated significantly under LA stresses as well when glucose and XOS were supplied, however, mannose transporter *manXYZ* (Figure 5D) was significantly up-expressed in LAC when compared to NC and maintained its transcription level in XOS but not in glucose. Moreover, xylose isomerization (Figure 6A), two glycolysis units (Figure 6B), and galactose metabolism (Figure 6C) were significantly up-regulated by XOS, compared to LAC. Although, the arginine deiminase system (ADS) (Figure 6D) was not highly regulated in fold change measurements, when the immense overall transcriptional activities of ADS were taken into consideration (Supplementary Tables 1, 2), the high expression level in XOS of this secondary metabolic pathway should be noticed.

### RT-qPCR Analysis

**FIGURE 7:**
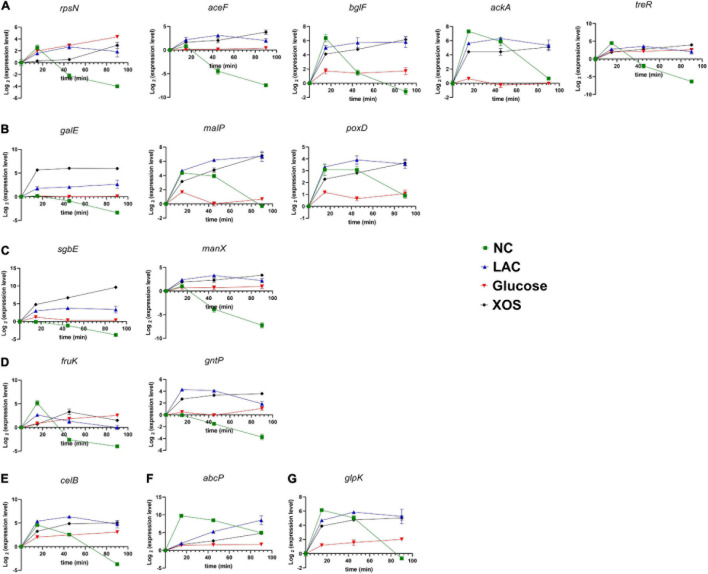
Genes regulation by lactic acid stress and carbohydrate supplementation evaluated using RT-qPCR. Genes were categorized by their transcriptional regulator families: no regulator **(A)**, LacI **(B)**, GntR **(C)**, DeoR **(D)**, HxlR **(E)**, PhoP **(F)**, LysR **(G)**. NC, neutral control without carbohydrate supplement; LAC, lactic acid control of pH 4.2; Glucose, LAC supplied with glucose; XOS, LAC supplied with XOS.

In accordance with global transcriptome analysis, a total of 15 genes were selected for follow-up reverse-transcription quantitative real-time PCR (RT-qPCR) experiments. In this section, transcriptional activities of *P. pentosaceus* were terminated at 15 min, 45 min (RNA-seq sampling time), as well as 90 min and were subjected to RT-qPCR analysis (Figure 7). In contrary to a transcriptome snapshot in RNA-seq, this analysis highlighted the transcriptional regulation progression during culture incubation. Firstly, most genes experienced up-expression at the 15 min time point compared to their original transcription level (time zero), regardless of the culture conditions. It is worth notice that, at 15 min, the NC culture that carries neither LA stress nor extra carbohydrate content also led to gene up-expression, which caused relative high expression level, for instance, *bglF*, *ackA*, and *abcP*. However, these up-expression faded away at 45 min and 90 min as the gene expression level of NC dropped dramatically for almost all the 15 selected genes.

Interesting, comparable to the results in RNA-seq (45 min of incubation), most down-regulated genes in glucose group were not highly expressed, especially at 15 min, such as *bglF*, *ackA*, *galE*, *malP*, and *glpK*. Furthermore, the genes were categorized according to the transcriptional regulator families e.g., no regulator (Figure 7A), LacI (Figure 7B), GntR (Figure 7C), DeoR (Figure 7D), HxlR (Figure 7E), PhoP (Figure 7F), LysR (Figure 7G), since many of these genes located in a polycistronic unit. The expression of the genes was not solely decided by the regulators as the distinctive gene expression patterns discovered within a regulator family, such as *galE* and *malP; sgbE* and *manX.* Nevertheless, identical gene expression patterns were observed in *malP* and *poxD*, both were regulated by regulator of LacI family.

## Discussion

When applied as industrial fermentation factories or in fermented foods, LAB inevitably encounter organic acid stresses, which are primarily originated from LA (Wang et al., 2018). Many methodologies have been employed to combat the challenges induced by LA stress as well as to improve innate LA resistance in LAB, such as intracellular microenvironment and cell membrane engineering, adaptive evolution, and extracellular protective supplementation (Zhang et al., 2012; Wu et al., 2014; Bucka-Kolendo and Sokołowska, 2017). Also, owing to their broad utilization and perceived host beneficial effects in foods (Mathur et al., 2020), modern applications of LAB in foods request sustainable bacterial survivability and viability in both food matrix and host GI track, which, unsurprisingly, ask for solid acid resistance from the feature LAB strains. The connection between acidic resistance of LAB and the presence of metabolizable carbohydrates have been previously established (Corcoran et al., 2005). *P. pentosaceus* has long been engineered and applied as bacterial factory to produce valuable substances (Wu et al., 2004; Sun et al., 2019). More and more recent studies have illustrated that *P. pentosaceus* could exert comprehensive host beneficial effects, e.g., antioxidant activities (Zhang et al., 2020), hyperlipidemia relief (Wang et al., 2020), and gut microbiota restoration (Hao et al., 2021). Moreover, the combination between beneficial bacteria (probiotic) and fermentable non-digestible dietary carbohydrate (prebiotic) could manifest synergistic health improvement effects, thus has been attracted much attention in novel functional food manufacturing (Swanson et al., 2020).

The results in this study demonstrated that LA and AA possessed similar growth retard and population viability reduction capability on *P. pentosaceus* strains (Figures 1, 2 and Supplementary Figures 1, 2), although the effectiveness may vary owing to their different acidic levels. In agreement with previous research (Corcoran et al., 2005), the addition of utilizable carbohydrates increased the organic acid resistance for all surveyed groups, whereas XOS exhibited the highest population protective effects when pH is low (pH = 4.2). This finding is rather interesting when consider that previous study found XOS was the least metabolizable carbohydrate for *P. pentosaceus* when pH value was close to neutral (Han et al., 2021). Further experiments highlighted that the viability protective effect of XOS is independent from its monosaccharide component, whereas xylose did not achieve comparable results (Figures 2B,D and Supplementary Figure 2). The authors propose that the combination between XOS and *P. pentosaceus* may have application potential in both fermented food and industrial fermentations.

High-throughput sequencing and its everlasting advancement has empowered researchers to conducted high-resolution global transcriptome studies (Reiter, 2021). RNA-seq pipeline in this study reliably captured the mRNA expression profile. The experiment was designed to discover the LA stress caused transcriptional regulations as well as the regulations induced glucose and XOS under LA stress. As presented, LA stress imposed extensive gene regulations in *P. pentosaceus*, whereas fewer genes were mobilized in glucose and XOS utilization under LA conditions (Figures 3, 4). Total of 259 genes were significantly up- or down expressed in LAC compared to NC, which accounted 16.5% of 1572 mRNA coding sequences (CDs). As a self-imposed stress which is often wielded by the LAB to achieve ecological competitiveness, *P. pentosaceus* displayed dynamic transcriptional regulation capacities to LA stresses, although prolonged exposure could also lead to cell death (Papadimitriou et al., 2016). Furthermore, glucose repressed the expression of 83 genes but promoted only 2, this finding aligns with general knowledge that glucose tends to suppress multiple metabolic pathways in LAB. this can be attributed to the fact that glucose is highly metabolizable that only requires the mobilization of limit gene repertoires (Gänzle and Follador, 2012; Goh and Klaenhammer, 2015). On the contrary, XOS supplementation resulted in the up-expression of 19 genes.

The pathways illustrated in detail consist of the most highly regulated genes in XOS metabolism of *P. pentosaceus* (Figures 5, 6). Interestingly, phosphate ABC transporter *pstSCAB* is heavily down-regulated in LA stress and further down-regulated in glucose and XOS environment (Figure 5C). The regulation of this transporter is often up-expressed during phosphate insufficiency or starvation (Vuppada et al., 2018), but not in LA stress, apparently. The expression of *manXYZ* operon, responsible for multiple of sugar transportations (Okochi et al., 2007), is higher expressed in LA stress and XOS culture, but significantly down-regulated in glucose-supplied LA stress compared to LAC (Figure 5D). When XOS is supplied to LA stressed *P. pentosaceus*, the pathways that carry out the biofunction of xylose isomerization and metabolism (Figure 6A) were significantly up-regulated for energy production, presumably. The XOS catabolic pathway in LAB has been previously proposed, XOS (degree of polymerization: 2−6) were carried by ABC transporters and hydrolyzed either by endo-1,4-β-xylanase and β-xylosidase to release xylose which further participates monosaccharide metabolism (Goh and Klaenhammer, 2015). Notably, the glycolysis unit 1 (Figure 6B) that included two fructose phosphorylases, T256_00805 and T256_00800, was significantly down-regulated by LA stress, while glycolysis unit 2 (T256_05730 and T256_05735) was up-regulated by LA stress and further up-expressed with the addition of XOS. Also, the ADS, previously known to be involved in acidic environment response (Liu et al., 2008), was mildly down-regulated by LA stress alone, but apparently further down-regulated by the addition glucose. This finding is expected since glucose, as an efficient energy supplier, often suppress the expression of this secondary ATP producing pathway (Hitzmann et al., 2013).

Regulation of gene expression is of great importance for overall bacterial fitness and whether can respond to environmental stresses at a high pace is truly a matter of life and death to bacterial cells (Browning and Busby, 2016). This study selectively evaluated the gene transcription of 15 genes during the 90-min of incubation time to profile the regulation pattern of the LA stress, glucose and XOS to *P. pentosaceus*. Firstly, when neither LA stress nor no additional carbohydrate is supplied in the mMRS culture, initial transcriptional response (often up-expression) for the bacterial still occurred at 15 min and then fadeaway after 90 min. This phenomenon may be owing to the adopted culture re-introduction methodology for the transcriptome experiment since all medium remained separated from their aimed bacterial cells prior to time 0. Moreover, glucose supplementation could rapidly achieve its transcriptional regulation effect (often suppression) as the expression level often remain stable for glucose post the 15-min time point. This finding could be attributed to the high glucose uptake efficiency in bacteria (Jahreis et al., 2008). Furthermore, from the transcriptional promoter classification, it can be inferred that the expression of the genes was not solely decided by these promoters, whereas only the LacI family has similar gene transcriptional patterns observed in their downstream polycistrons (*malP* and *poxD*). It is also worth noting that very similar patterns were discovered between gene duos with and without a transcriptional regulator (Supplementary Table 4), such as *fruK* (DeoR) and *treR*, *manX* (GntR) and *aceF*, and *glpK* (LysR), and *ackA*, which implying RNA polymerase-centered transcriptional regulation, instead of promoter-centered regulation, is the predominant transcription regulatory mode in LA stress response and respective carbohydrate metabolisms.

## Conclusion

When applied as environmental stresses, LA and AA possessed similar growth stagnate effect and eventually lead to cell death in *P. pentosaceus*. The supplementation of carbohydrate and functional oligosaccharides, especially XOS, could protect bacterial culture and retain the culture viability. RNA-seq analysis revealed extensive global transcriptional regulation caused by LA stress while XOS supplementation maintained most regulations. However, glucose suppressed numerous genes when supplied as energy source under LA stress. Follow-up RT-qPCR survey illustrated that RNA polymerase-centered transcriptional regulation should be considered as the primary gene regulation approach for *P. pentosaceus* under LA stress. Alone with previous literatures, this study reiterates that the combinations between *P. pentosaceus* and functional oligosaccharides has the potential to be applied under acidic environment, e.g., dairy foods and acidic fermentations. Also, when LAB, like *P. pentosaceus*, are applied in fermentation cultures, the carbohydrate formulation should be thoughtfully evaluated and modified to ensure desirable fermentation results.

## Data Availability Statement

Underlying RNA sequencing data in FASTQ format have been deposited into NCBI database and are available under the Bio Project accession number of PRJNA678704.

## Author Contributions

DH carried out the investigation and drafted the original manuscript. QY contributed to the methodology and designed the experiments. JL analyzed the data and revised the manuscript. ZJ supervised the project and reviewed the manuscript. SY supervised the experiments and edited the manuscript. All approved the submitted version of the article.

## Conflict of Interest

The authors declare that the research was conducted in the absence of any commercial or financial relationships that could be construed as a potential conflict of interest.

## Publisher’s Note

All claims expressed in this article are solely those of the authors and do not necessarily represent those of their affiliated organizations, or those of the publisher, the editors and the reviewers. Any product that may be evaluated in this article, or claim that may be made by its manufacturer, is not guaranteed or endorsed by the publisher.

## Footnotes

1
http://usegalaxy.org

2
https://img.jgi.doe.gov/m/

3
https://pathways.embl.de/

4
https://www.ncbi.nlm.nih.gov/tools/primer-blast/
