# Supplementary material for: Transcriptomic Analysis of Pediococcus pentosaceus Reveals Carbohydrate Metabolic Dynamics Under Lactic Acid Stress
Source: Front Microbiol. 2021 Sep 16;12:736411. doi: 10.3389/fmicb.2021.736411 (PMC8481956; doi:10.3389/fmicb.2021.736411)
Supplement: Supplementary file 3 [file Data_Sheet_1.docx]

**Transcriptomic Analysis of *Pediococcus pentosaceus* Reveals Carbohydrate Metabolic Dynamics under Lactic Acid Stress**

**Supplementary Material**

Dong Han^1,2^, Qiaojuan Yan^3^, Jun Liu^1^, Zhengqiang Jiang^1*^, Shaoqing Yang^1*^

^1^Key Laboratory of Food Bioengineering (China National Light Industry), College of Food Science and Nutritional Engineering, China Agricultural University, Beijing, China

^2^School of Food and Health, Beijing Technology and Business University, Beijing, China

^3^College of Engineering, China Agricultural University, Beijing, China

* Correspondence:
Shaoqing Yang (ysq@cau.edu.cn) and Zhengqiang Jiang (zhqjiang@cau.edu.cn)

Keywords: *Pediococcus pentosaceus*, lactic acid stress, carbohydrate metabolism, functional oligosaccharides, RNA-seq

**Running title:** RNA-seq of *Pediococcus pentosaceus*

**TABLE S3.** Composition of modified de Man, Rogosa and Sharpe (mMRS) medium

| **Ingredient concentration: g/L** | | | |
| --- | --- | --- | --- |
| Oligosaccharides | 20.0 | Peptone | 10.0 |
| Beef extract | 5.0 | Yeast extract | 4.0 |
| Sodium acetate trihydrate | 5.0 | Polysorbate 80 | 1.0 |
| Dipotassium hydrogen phosphate | 2.0 | Triammonium citrate | 2.0 |
| Magnesium sulfate heptahydrate | 0.2 | Manganese sulfate tetrahydrate | 0.05 |

**TABLE S4** Comprehensive information of selected genes in RT-qPCR study. N/A: not appliable.

| **CDS tag** | **Gene function** | **Regulator family** | **Gene name** | **Primer direction** | **Primer name** | **Primer sequence** | **Amplicon size** |
| --- | --- | --- | --- | --- | --- | --- | --- |
| T256_00115 | PTS system, cellobiose-specific IIC component | HxlR | *celB* | forward | celB_F | AAGCACTTGGAAAGGCAACTT | 95 |
|  |  |  |  | reverse | celB_R | TGGAACCGGTGCAACCATTAT |  |
| T256_00950 | L-ribulose 5-phosphate 4-epimerase | GntR | *sgbE* | forward | sgbE_F | CCGCCGCAGAAATTGATGTA | 87 |
|  |  |  |  | reverse | sgbE_R | TAGTGAGTGCCGGAGTTACC |  |
| T256_01160 | UDP-galactose 4-epimerase | LacI | *galE* | forward | galE_F | TGGTCAGATGTGGCTGATGG | 92 |
|  |  |  |  | reverse | galE_R | TGGTCTTCCCCAATACTGCC |  |
| T256_02280 | Phosphate ABC transporter membrane protein 1, *PhoT* family | PhoP | *abcP* | forward | abcP_F | CAGGAAAACGTGGGCGTAAA | 95 |
|  |  |  |  | reverse | abcP_R | GACGACCGTCAACCCGATAA |  |
| T256_04730 | Maltose phosphorylase | LacI | *malP* | forward | malP_F | TGTTGCCGTTCTACTGACGA | 93 |
|  |  |  |  | reverse | malP_R | GCGGTGGACACTCCGTTAT |  |
| T256_05735 | Fructose-1-phosphate kinase | DeoR | *fruK* | forward | fruK_F | CACCTTCACCAGCCATTGAAA | 95 |
|  |  |  |  | reverse | fruK_R | GGAAGTAACCACCGATGCAGA |  |
| T256_06175 | Pyruvate oxidase | LacI | *poxD* | forward | poxD_F | GAGGTCCATCTGTCCGATTCA | 87 |
|  |  |  |  | reverse | poxD_R | CGCGTCCATTCTACAAAGCG |  |
| T256_06715 | Gluconate permease | DeoR | *gntP* | forward | gntP_F | ACACCGTCGTGGACAATCAA | 92 |
|  |  |  |  | reverse | gntP_R | CCGGGATTCGGTATTTCGGT |  |
| T256_06945 | SSU ribosomal protein S14P | N/A | *rpsN* | forward | rpsN_F | ACGGTATACAGAGTGTGGACG | 85 |
|  |  |  |  | reverse | rpsN_R | TGTAAAGAGTGAACGTCCTGCT |  |
| T256_07500 | PTS system D-glucose-specific IIA component, Man family | GntR | *manX* | forward | manX_F | GTCGTCTTTGTCCAAGCTGT | 88 |
|  |  |  |  | reverse | manX_R | GTCGTCCAGGACACTTCGATT |  |
| T256_07985 | Glycerol kinase | LysR | *glpK* | forward | glpK_F | TCTCGGCAACCAAAATTCGC | 85 |
|  |  |  |  | reverse | glpK_R | GTTCCAAACAACAGCTCGCC |  |
| T256_08725 | Dihydrolipoamide acetyltransferase | N/A | *aceF* | forward | aceF_F | GTCGTCTTTGTCCAAGCTGT | 88 |
|  |  |  |  | reverse | aceF_R | GTCGTCCAGGACACTTCGATT |  |
| T256_08895 | PTS system IIA component, *Glc* family | N/A | *bglF* | forward | bglF_F | ACGATTTTCCAGCCAACGGA | 92 |
|  |  |  |  | reverse | bglF_R | GGCAAAACGCATTGGTGGAG |  |
| T256_08900 | Transcriptional regulator, *GntR* family | N/A | *treR* | forward | treR_F | CCAGTTTCCGGTATCAAATCGT | 95 |
|  |  |  |  | reverse | treR_R | GGCACCAAACGAGGCTCTAA |  |
| T256_08980 | Acetate kinase | N/A | *ackA* | forward | ackA_F | ATGCCCCACTTCATAACCCG | 94 |
|  |  |  |  | reverse | ackA_R | GAAGTGTCGAAAACCGCCAC |  |
|  |  | N/A | 16s rDNA | forward | 16srDNA_F | ACATTGGGACTGAGACACGG | 86 |
|  |  |  |  | reverse | 16srDNA_R | GTTGCTCCATCAGACTTGCG |  |


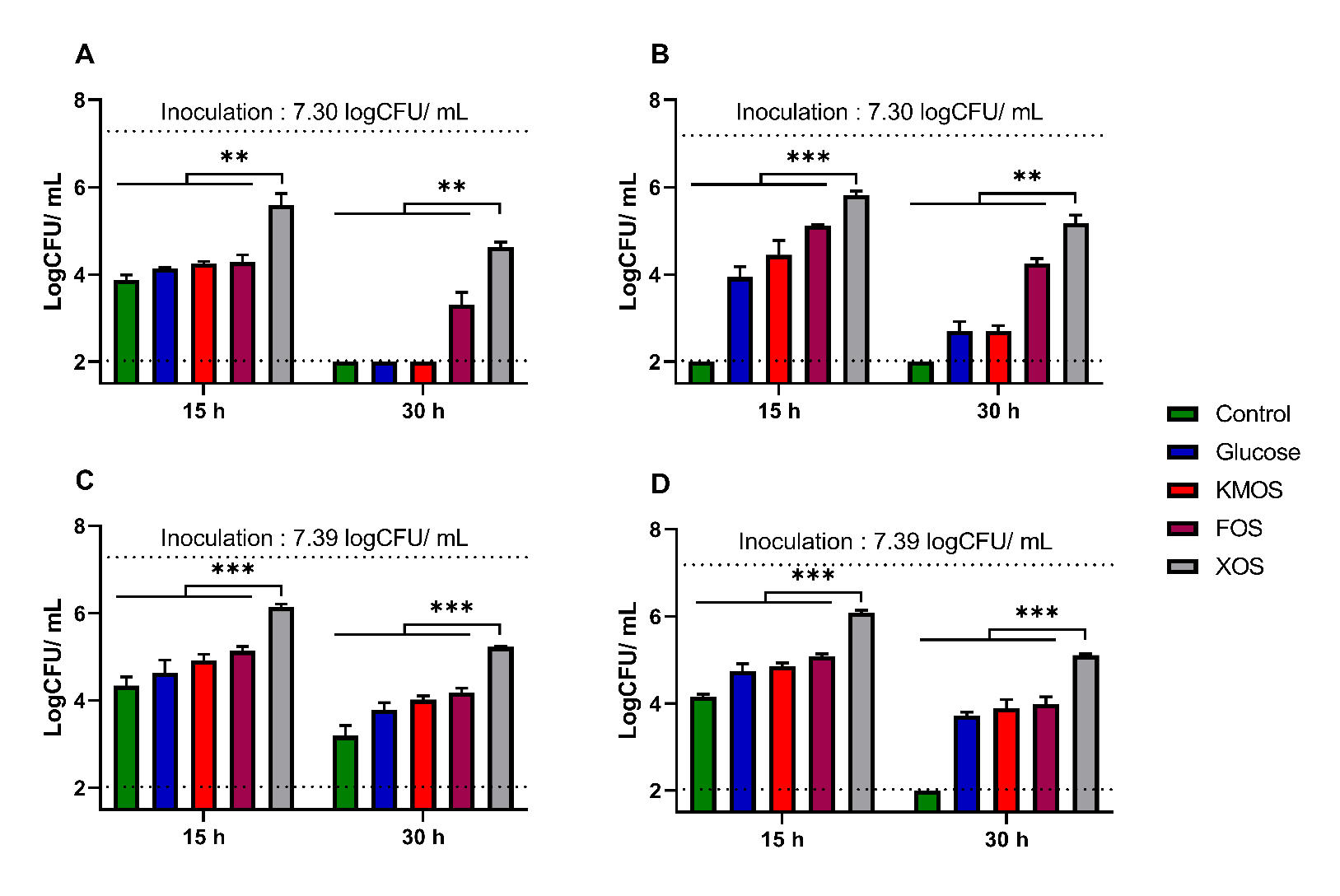


**FIGURE S1** Remaining population in culture of pH 4.2 measured by plate count method for *P. pentosaceus* CGMCC 1.2441 adjusted using lactic acid (A) or acetic acid (B) and *P. pentosaceus* CGMCC 1.10999 adjusted using lactic acid (C) or acetic acid (D). (*: *P* < 0.05; **: *P* < 0.01; ***: P < 0.001). Detect limitation: 2 log CFU/ ml.


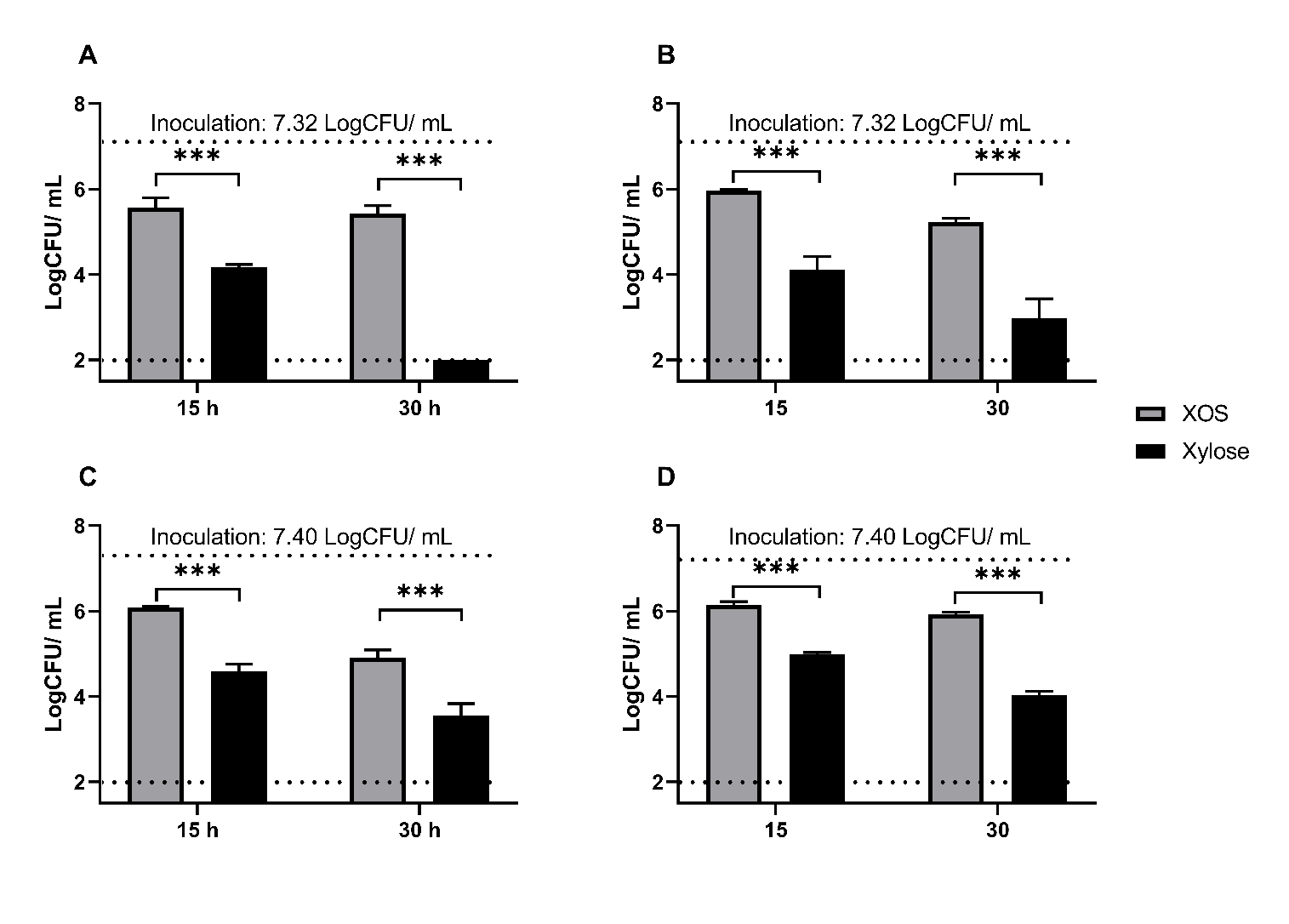


**FIGURE S2** Remaining population in XOS or xylose culture of pH 4.2 measured by plate count method for *P. pentosaceus* CGMCC 1.2441 adjusted using lactic acid (A) or acetic acid (B) and *P. pentosaceus* CGMCC 1.10999 adjusted using lactic acid (C) or acetic acid (D). (*: *P* < 0.05; **: *P* < 0.01; ***: P < 0.001). Detect limitation: 2 log CFU/ml.


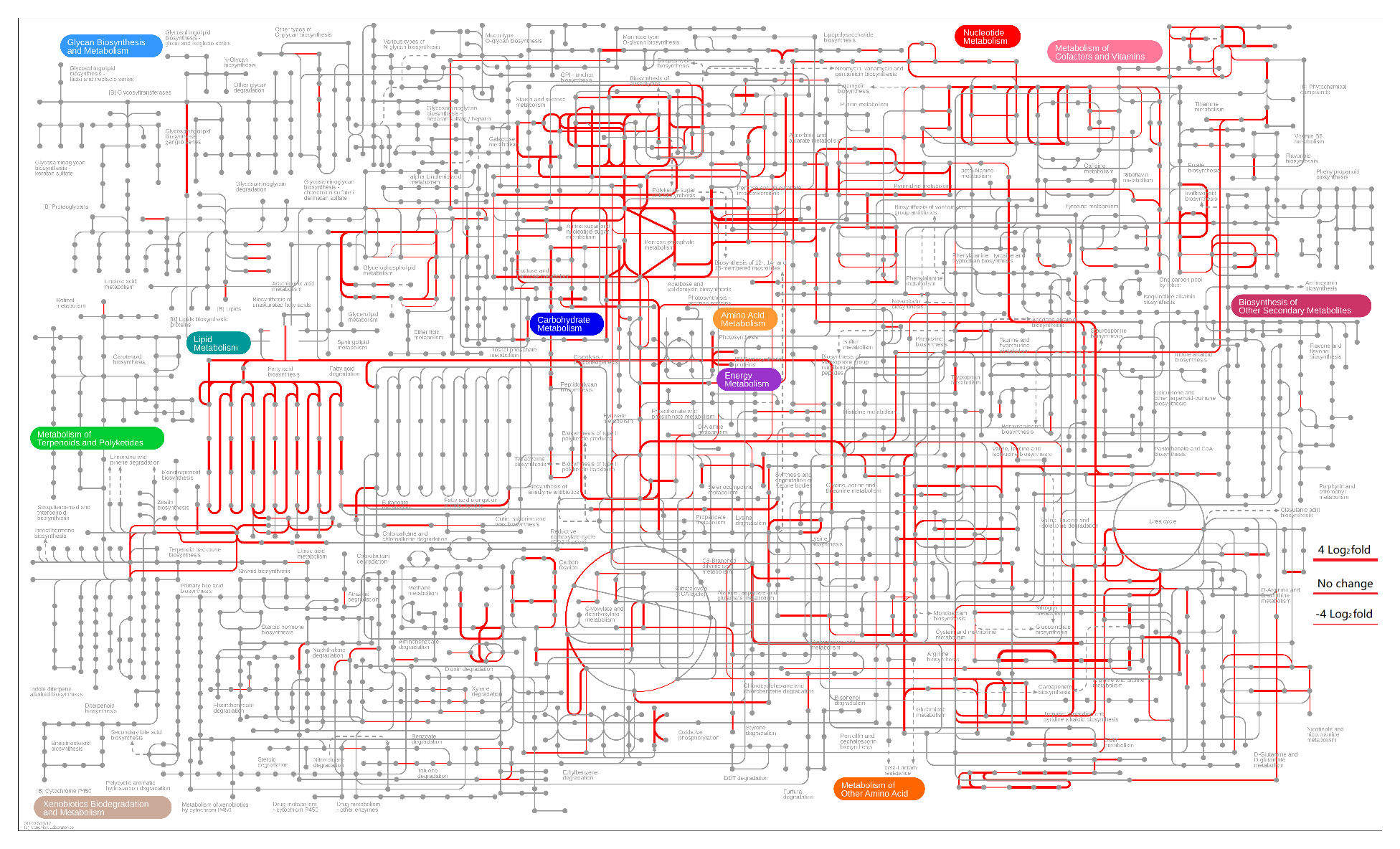
**FIGURE S3 Global transcriptomic expressions for LAC compared to NC*.*** Metabolic pathways were reconstructed on respective Clusters of Orthologous Groupsof proteins (COGs) by iPath 3 on Log2 (expression level) of each metabolic pathway are displayed on proportion to their line width.
